# Supplementary material for: Isogenic human pluripotent stem cell pairs reveal the role of a KCNH2 mutation in long-QT syndrome
Source: EMBO J. 2013 Nov 8;32(24):3161–75. doi: 10.1038/emboj.2013.240 (PMC3981141; doi:10.1038/emboj.2013.240)
Supplement: Supplementary Information [file emboj2013240s1.pdf]

Supplementary Information

This appendix has been provided by the authors to give readers additional information about their work.

Supplement to: Bellin, et al. Isogenic human pluripotent stem cell pairs reveal the role of a KCNH2 mutation in long-QT syndrome

Supplementary Table S1. Primers used for genotyping, screening for targeted clones, and for qRT-PCR.

**Gene**	**Use**	**Primer forward**	**Primer reverse**
KCNH2 (genotyping)	Sequencing	ATGGAGGACTGCGAGAAGAG	TACCTGAGAAAGCGAGTCCA
KCNH2 (a+b)	Targeting screening	CTGGGTTCTGTACGCTCCTG	CACCTTCCAGCTCCACTCTC
KCNH2 (c+d)	Targeting screening	AGACTATGGCGTGTGTGGTT	GGGCTGACCGCTTCCTCGTGC
KCNH2 (neo excised)	Neo excision test	TCGATGGATGCAGAGTGTGC	CACCTTCCAGCTCCACTCTC
KCNH2 (exon1)	Sequencing	CCGAAGCCTAGTGCTGGGCC	ATCCACACTCGGAAGAGCTC
KCNH2 (exon2)	Sequencing	GGCTGTGTGAGTGGAGAATG	ATCTCCCAGACCTGTCACCT
KCNH2 (exon3)	Sequencing	ATCATAGCCAGCGTGAGAAA	CCAAAGAAATGAGACCACGA
KCNH2 (exon4)	Sequencing	GGTGTGAGAGACAGGGATGA	TAATAGCGCAACAAGCCACT
KCNH2 (exon5)	Sequencing	TGCTTCCTTAGAGTGGGACA	CTTGGCCTGGAAGAATTAGG
KCNH2 (exon1b)	Sequencing	TCCTCCCTTGCTTCTCTCTT	TCCCAAAGCTTCCTACTTCC
KCNH2 (exon6)	Sequencing	AACTGTTTGGAGCCAGTCCT	GAGGAGCTTGTGTGGAGAGA
KCNH2 (exons7-8)	Sequencing	CACCTCTTAGGAGGAGGGTCT	GCCTGAGACTTGTTTGCTGT
KCNH2 (exon9)	Sequencing	TGGGATGGTGGAGTAGAGTG	CACATGGCCCTTAGTGAAAC
KCNH2 (exons10-11)	Sequencing	GTGATTGGCTAAGAGGGTGTT	GGCCCTCCTTTGTTCTATGT
KCNH2 (exons12-13-14)	Sequencing	CTTCCTGCCCAGTCCTCTCT	GTCTCGGGCTCAGTCAGTTC
KCNH2 (exon15)	Sequencing	CCACCTGACTCTCCTCTTGG	AGGAACTGGGTGAGGGAGAC
MYC endogenous	qRT-PCR	AGAAATGTCCTGAGCAATCACC	AAGGTTGTGAGGTTGCATTTGA
KLF4 endogenous	qRT-PCR	ATAGCCTAAATGATGGTGCTTGG	AACTTTGGCTTCCTTGTTTGG
OCT4 endogenous	qRT-PCR	GACAGGGGGAGGGGAGGAGCTAGG[1]	CTTCCCTCCAACCAGTTGCCCCAAAC[(1)]
SOX2 endogenous	qRT-PCR	GGGAAATGGGAGGGGTGCAAAAGAGG[1]	TTGCGTGAGTGTGGATGGGATTGGTG[(1)]
LEFTYA	qRT-PCR	TACAGGTGTCGGTGCAGAGG	ATTGGTGCTTCAGGGTCACA
NANOG	qRT-PCR	TGCAAGAACTCTCCAACATCCT	ATTGCTATTCTTCGGCCAGTT
REX1	qRT-PCR	ACCAGCACACTAGGCAAACC	TTCTGTTCACACAGGCTCCA
TDGF1	qRT-PCR	CCCAAGAAGTGTTCCCTGTG	ACGTGCAGACGGTGGTAGTT
MYC transgene	qRT-PCR	GGAAACGACGAGAACAGTTGA	CCCTTTTTCTGGAGACTAAATAAA[(1)]
KLF4 transgene	qRT-PCR	CCACCTCGCCTTACACATGA	CCCTTTTTCTGGAGACTAAATAAA[(1)]
OCT4 transgene	qRT-PCR	GCTCTCCCATGCATTCAAAC	TTATCGTCGACCACTGTGCTGCTG[(1)]
SOX2 transgene	qRT-PCR	GGCCATTAACGGCACACTG	CCCTTTTTCTGGAGACTAAATAAA[(1)]

PDX1	qRT-PCR	AAGCTCACGCGTGGAAAG	GGCCGTGAGATGTACTTGTTG
PTF1A	qRT-PCR	GGCCCAGAAGGTCATCATC	TAGGGGAGGGAGGCCATA
SOX7	qRT-PCR	TGAACGCCTTCATGGTTTG	AGCGCCTTCCACGACTTT
AFP	qRT-PCR	GTGCCAAGCTCAGGGTGTAG	CAGCCTCAAGTTGTTCCTCTG
CD31	qRT-PCR	ATGCCGTGGAAAGCAGATAC	CTGTTCTTCTCGGAACATGGA
DES	qRT-PCR	GTGAAGATGGCCCTGGATGT	TGGTTTCTCGGAAGTTGAGG
ACTA2	qRT-PCR	GTGATCACCATCGGAAATGAA	TCATGATGCTGTTGTAGGTGGT
SCL	qRT-PCR	CCAACAATCGAGTGAAGAGGA	CCGGCTGTTGGTGAAGATAC
MYL2	qRT-PCR	TACGTTCGGGAAATGCTGAC	TTCTCCGTGGGTGATGATG
MYH11	qRT-PCR	GGAGGCCAAGATTGCACAG	CAGCAAGATTTCCTTCAGCTTC
CDH5	qRT-PCR	GAGCATCCAGGCAGTGGTAG	CAGGAAGATGAGCAGGGTGA
KRT14	qRT-PCR	CACCTCTCCTCCTCCCAGTT	ATGACCTTGGTGCGGATTT
NCAM1	qRT-PCR	CAGATGGGAGAGGATGGAAA	CAGACGGGAGCCTGATCTCT
TH	qRT-PCR	TGTACTGGTTCACGGTGGAGT	TCTCAGGCTCCTCAGACAGG
GABRR2	qRT-PCR	CTGTGCCTGCCAGAGTTTCA	ACGGCCTTGACGTAGGAGA
TNNT2	qRT-PCR	AGCATCTATAACTTGGAGGCAGAG	TGGAGACTTTCTGGTTATCGTTG
KCNH2-1a	qRT-PCR	TGGGAGCTGCTTCCTATGTC	TCCTTCTCCATCACCACCTC
KCNH2-1b	qRT-PCR	AGGGAGCCAAGTCCTCCAT	TTGTACTCAGGCAGCACGTC
KCNE2	qRT-PCR	TGTGTGCAACCCAGAAGAGA	CTTCCAGCGTCTGTGTGAAA
KCNQ1	qRT-PCR	CGCCTGAACCGAGTAGAAGA	TGAAGCATGTCGGTGATGAG
KCNJ11	qRT-PCR	GACCCTTGGAGCAGTGTTGT	GCTTTATTGACAACGGAGAAGG
KCNJ12	qRT-PCR	TGGATCCTTTCCAGTTGGTG	CGGCTCCTCTTGAGTTCTATCTT
KCND3	qRT-PCR	CCAATTCTAACCTGCCAGCTAC	CTGCTTTCAAATTAAGGCTGGA
SCN5A	qRT-PCR	GAGCTCTGTCACGATTTGAGG	GAAGATGAGGCAGACGAGGA
CACNA1C	qRT-PCR	CAATCTCCGAAGAGGGGTTT	TCGCTTCAGACATTCCAGGT
GAPDH	qRT-PCR	TCCTCTGACTTCAACAGCGA	GGGTCTTACTCCTTGGAGGC

1. Takahashi, K., Tanabe, K., Ohnuki, M., Narita, M., Ichisaka, T., Tomoda, K., and Yamanaka, S. 2007. Induction of pluripotent stem cells from adult human fibroblasts by defined factors. *Cell* 131:861-872.

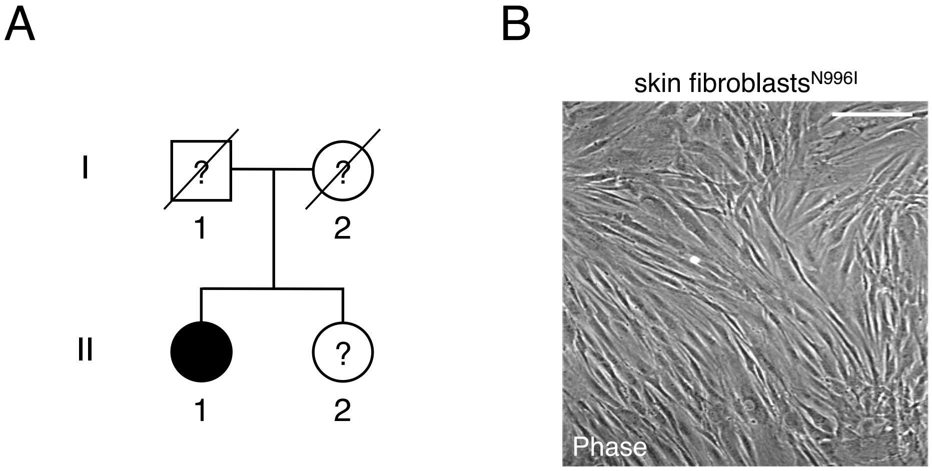

Supplementary Figure S1. Dermal fibroblasts from a patient with type-2 long-QT syndrome. (**A**) Family pedigree shows that patient II-1 is affected by LQT2. Squares indicate male family members, circles female family members, solid symbols family members with LQT2, open symbols with question mark genotype unknown. (**B**) Skin fibroblasts were grown from a skin biopsy from patient-II-1; scale bar: 200 μm.

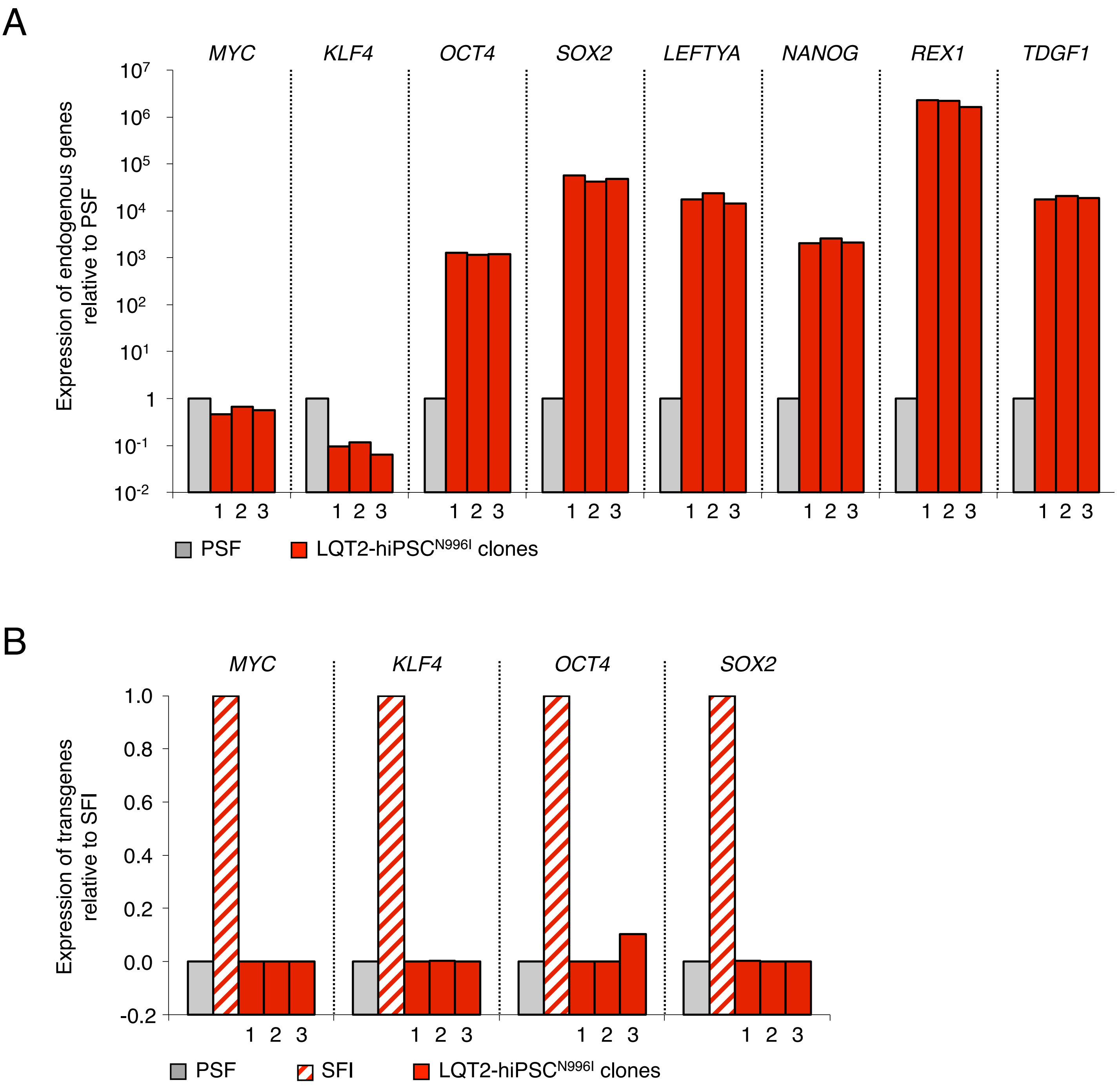

Supplementary Figure S2. Re-expression of endogenous genes associated with pluripotency and silencing of the transgenes in three LQT2-hiPSC^{N996I} clones. (**A**) qRT-PCR analysis of the endogenous pluripotency associated-genes (*MYC*, *KLF4*, *OCT4*, *SOX2*, *LEFTYA*, *NANOG*, *REX1*, and *TDGF1*) reveals similar activation of these genes in all three LQT2-iPSC^{N996I} clones (1, 2, and 3, red bars). Expression values are relative to the primary skin fibroblasts from which they were derived (PSF, grey bars). (**B**) qRT-PCR analysis of the four transgenes used for the reprogramming, *MYC*, *KLF4*, *OCT4*, and *SOX2*, in all three LQT2-hiPSC^{N996I} clones (1, 2, and 3, red bars). Transgenes silencing is demonstrated by expression levels similar to parental PSF (grey bar). Expression values are relative to skin fibroblasts after the retroviral transduction (SFI, red and white stripe bars). Expression values in panels A and B are normalized to *GAPDH*.

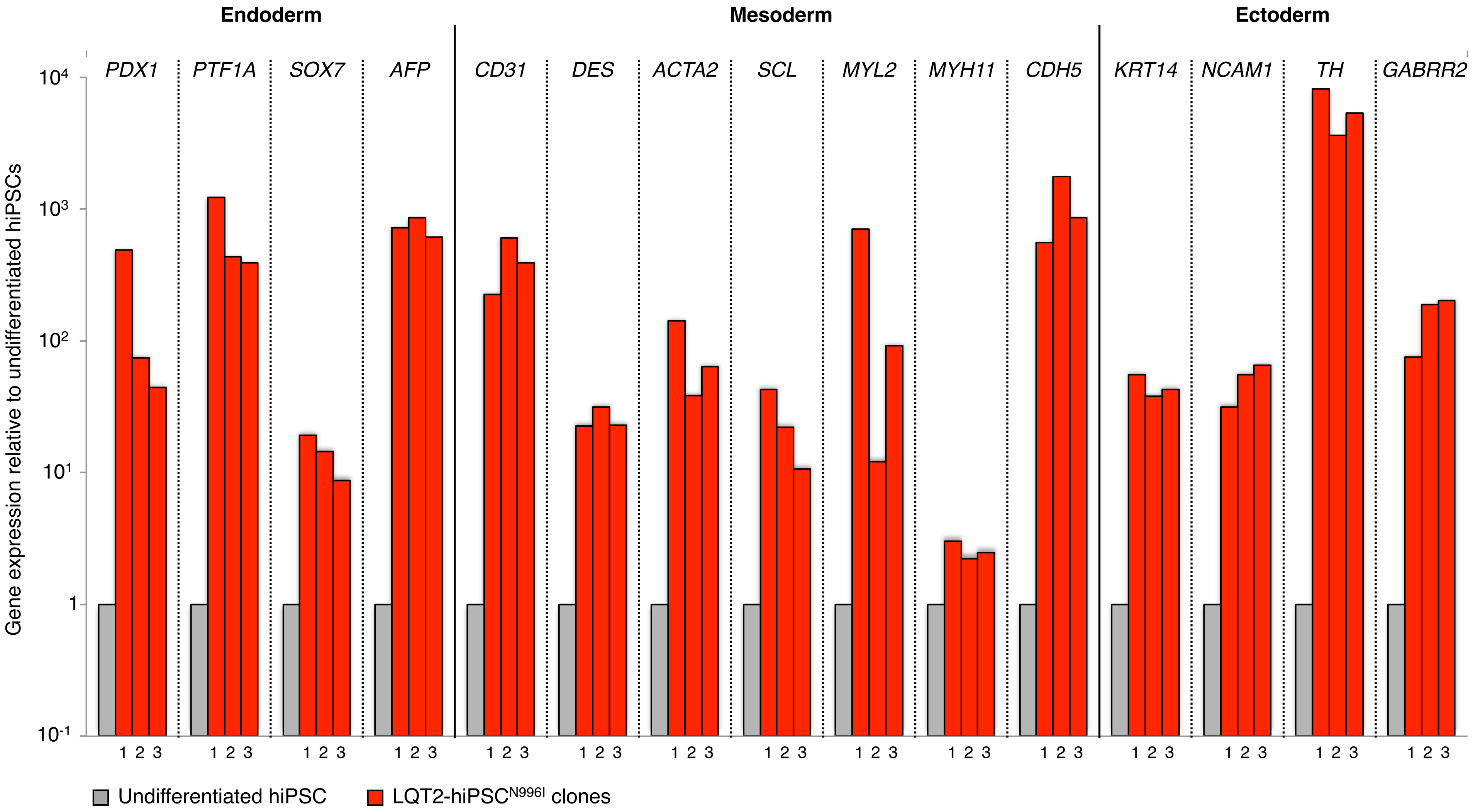

Supplementary Figure S3. Up-regulation of lineage markers representative of the three embryonic germ layers in three LQT2-iPSC^{N996I} clones at day 21 following spontaneous differentiation. The bar graph shows qRT-PCR analysis of markers of the three different germ layers, endoderm (*PDX1*, *PTF1A*, *SOX7*, and *AFP*), mesoderm (*CD31*, *DES*, *ACTA2*, *SCL*, *MYL2*, *MYH11*, and *CDH5*), and ectoderm (*KRT14*, *NCAM1*, *TH*, and *GABRR2*) in embryoid bodies at day 21 of *in vitro* differentiation from three LQT2-iPSC^{N996I} clones (1, 2, and 3, red bars). Gene expression values are relative to corresponding undifferentiated hiPSC clones (grey bar) and normalized to *GAPDH*.

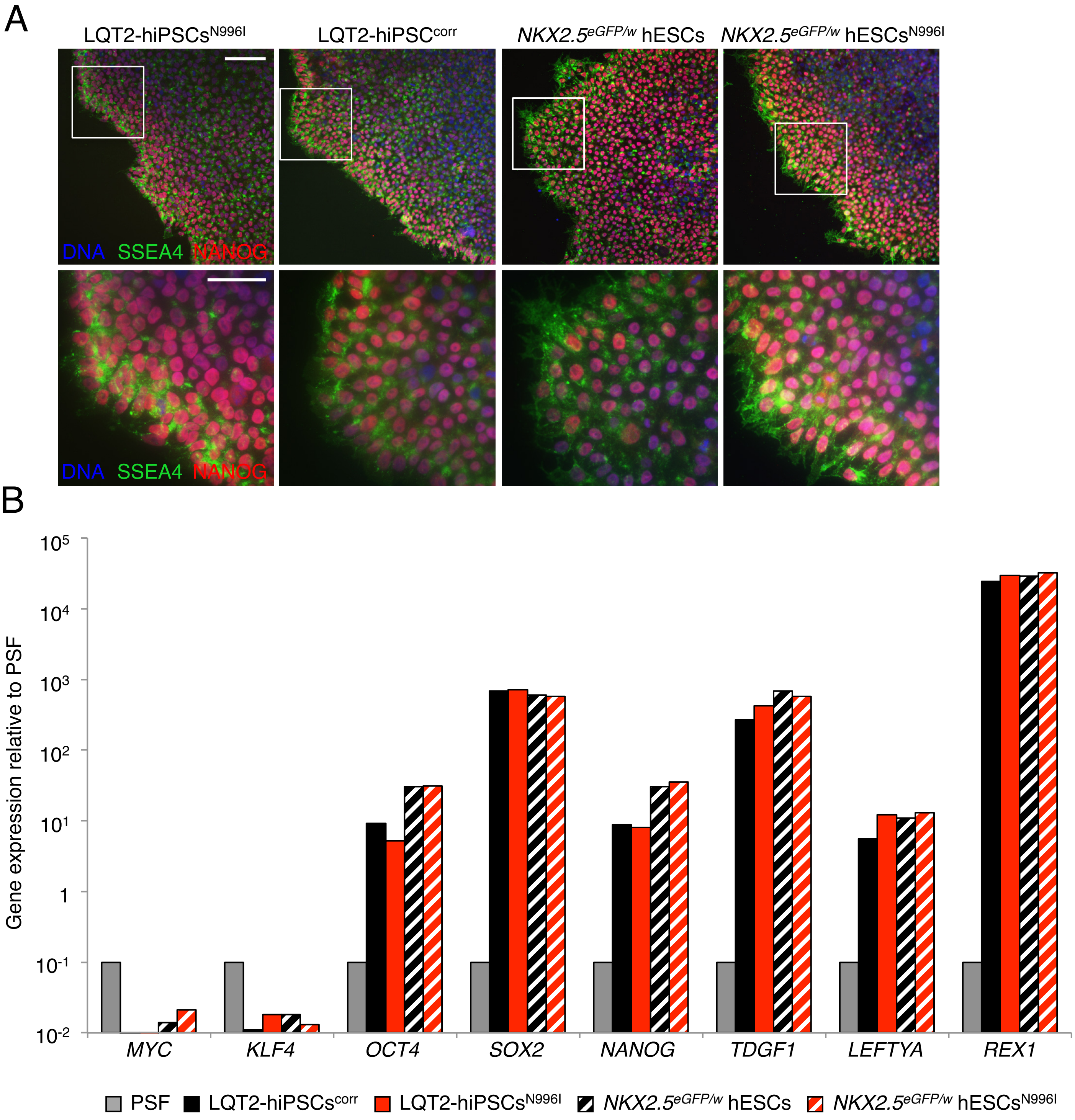

Supplementary Figure S4. Expression of endogenous genes associated with pluripotency in mutated and corrected hiPSCs (LQT2-hiPSCs^{N996I} and LQT2-hiPSCs^{corr}, respectively) and in wild-type and mutated hESCs (*NKX2.5*$^{eGFP/w}$ hESCs and *NKX2.5*$^{eGFP/w}$ hESCs^{N996I}, respectively). (**A**) Immunofluorescence analysis of pluripotency markers NANOG (red) and SSEA4 (green) and nuclear staining (DNA, blue). The lower panel images are a magnification of the area framed in the corresponding upper image. Scale bars: 100 μm (top panel), 50 μm (bottom panel) (**B**) qRT-PCR analysis of the endogenous pluripotency associated-genes (*MYC*, *KLF4*, *OCT4*, *SOX2*, *LEFTYA*, *NANOG*, *REX1*, and *TDGF1*) revealing similar expression of these genes in mutated and corrected hiPSCs and from wild-type and mutated hESCs. Expression values are relative to the primary skin fibroblasts from which LQT2-hiPSCs were derived (PSF, grey bars).

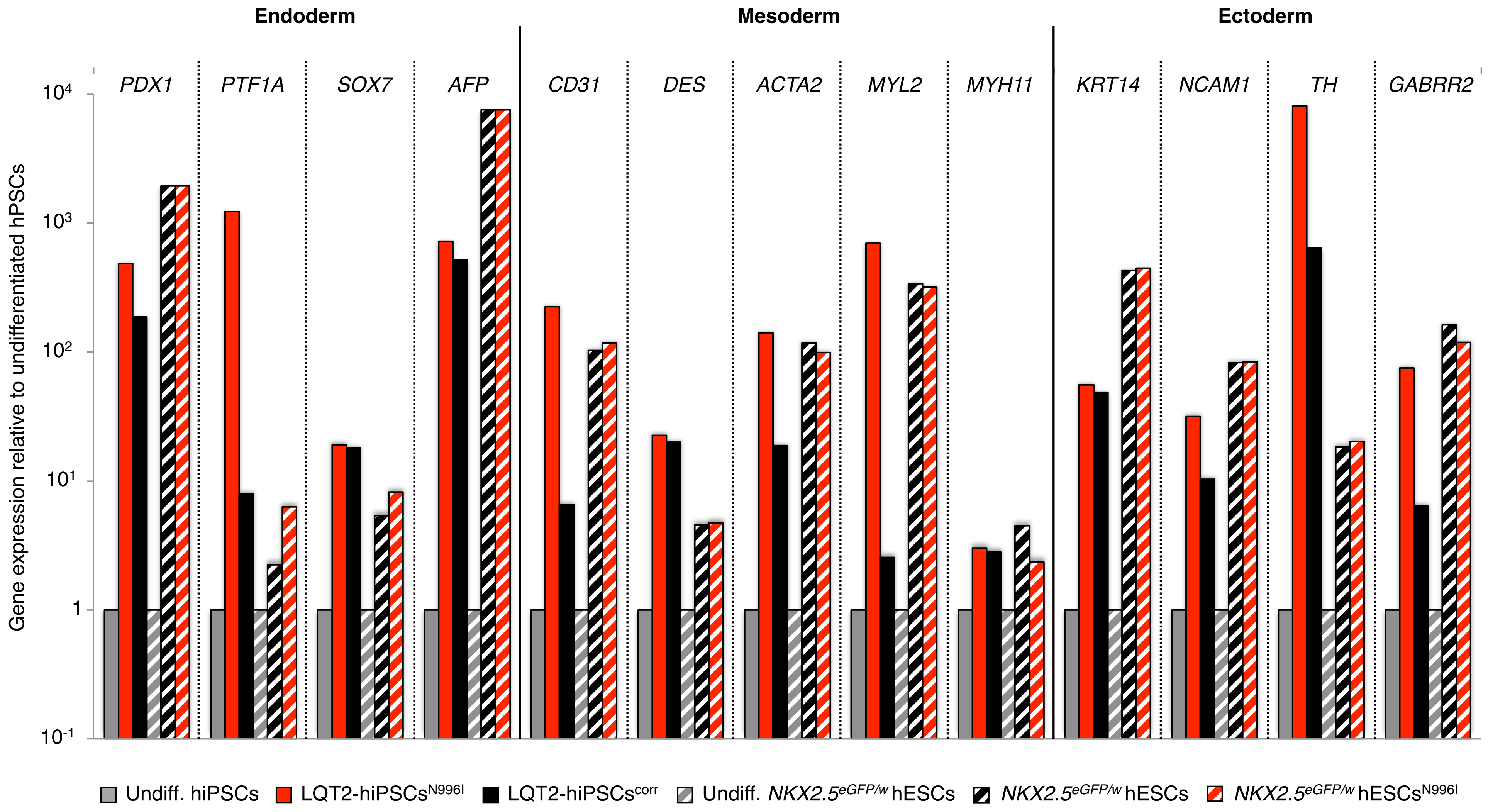

Supplementary Figure S5. Up-regulation of lineage markers representative of the three embryonic germ layers in the targeted human pluripotent stem cell (hPSC) lines at day 21 following spontaneous differentiation. The bar graph shows qRT-PCR analysis of markers of the three different germ layers, endoderm (*PDX1*, *PTF1A*, *SOX7*, and *AFP*), mesoderm (*CD31*, *DES*, *ACTA2*, *MYL2*, and *MYH11*), and ectoderm (*KRT14*, *NCAM1*, *TH*, and *GABRR2*) in embryoid bodies at day 21 of *in vitro* differentiation from LQT2-hiPSCs^{N996I} (red bars), the targeted LQT2-hiPSCs^{corr} (black bars), the *NKX2.5*$^{eGFP/w}$ hESCs (black and white striped bars), and the targeted *NKX2.5*$^{eGFP/w}$ hESCs^{N996I} (red and white striped bars). Gene expression values are relative to corresponding undifferentiated (Undiff.) hPSCs, LQT2-hiPSCs^{N996I} (grey bar) and *NKX2.5*$^{eGFP/w}$ hESCs (gray and white striped bars) and normalized to *GAPDH*.

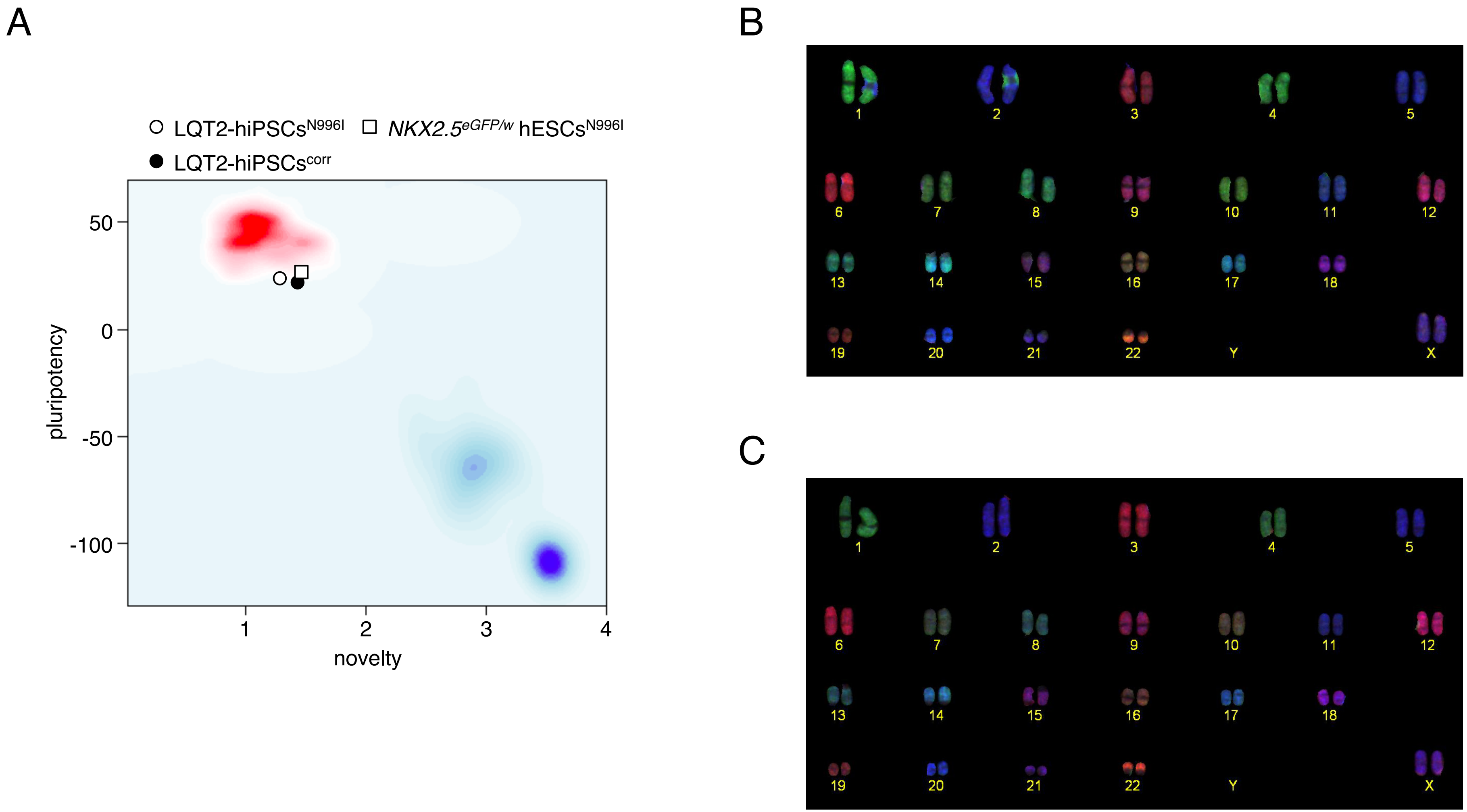

Supplementary Figure S6. Targeted LQT2-hiPSCs^{corr} and *NKX2.5*$^{eGFP/w}$ hESCs^{N996I} are pluripotent and maintain a normal karyotype. (**A**) PluriTest analysis of LQT2-hiPSCs^{N996I} (empty circle), LQT2-hiPSCs^{corr} (black circle), and *NKX2.5*$^{eGFP/w}$ hESCs^{N996I} (empty square). All tested lines have a high "pluripotency score" and a low "novelty score" indicating that they resemble normal human pluripotent stem cells (in red). (**B**) COBRA-FISH cytogenetic analysis of LQT2-hiPSCs^{corr} showing a normal 46 XX karyotype. (**C**) COBRA-FISH cytogenetic analysis of *NKX2.5*$^{eGFP/w}$ hESCs^{N996I} showing a normal 46 XX karyotype.

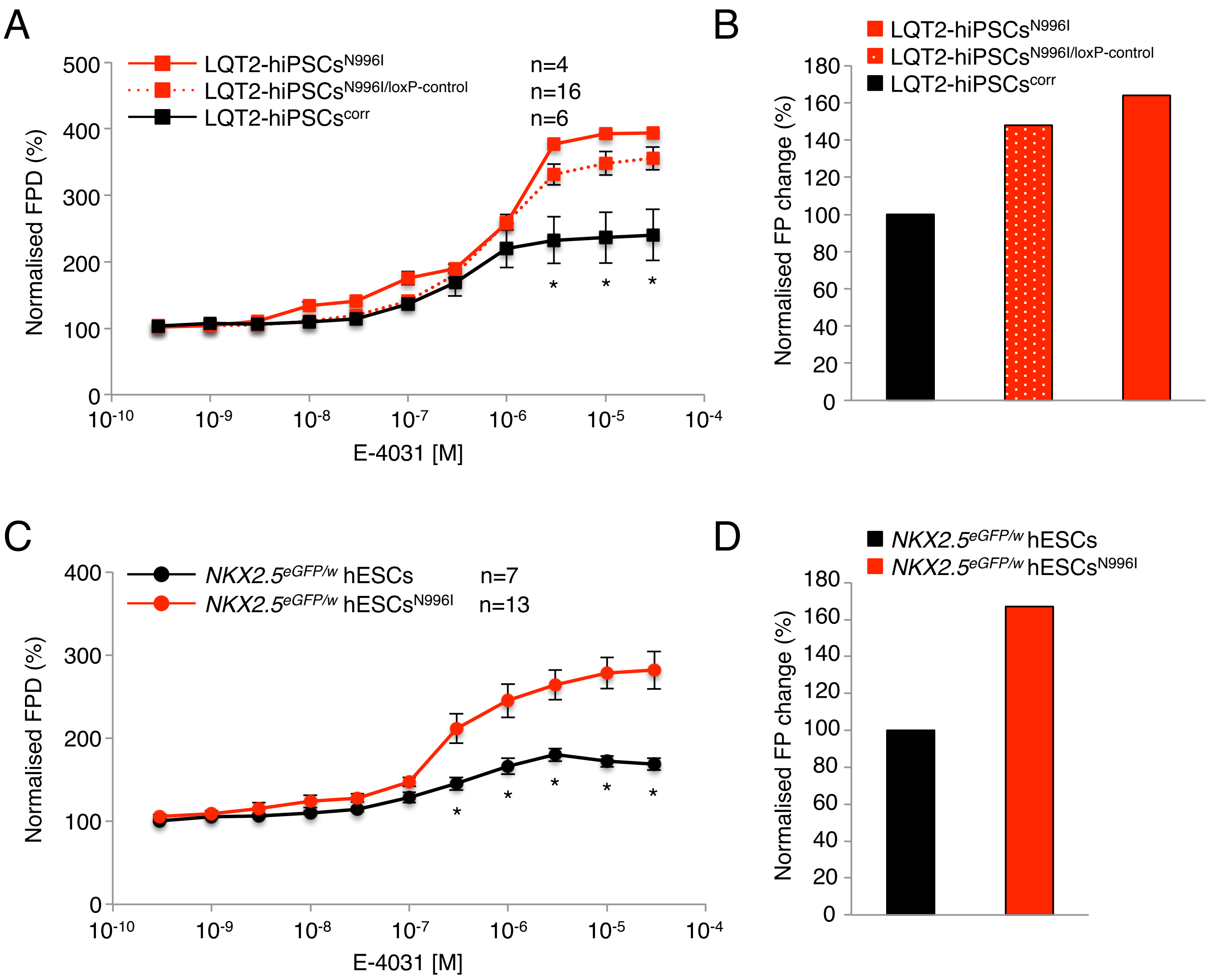

Supplementary Figure S7. The effect of I_{Kr} blockade induced by increasing amounts of E-4031 on cardiac repolarization in hPSC-derived CMs. (**A**) FPD dose response relationship in presence of increasing amounts of E-4031 in mutated (LQT2-hiPSCs^{N996I}), loxP-control (LQT2-hiPSCs$^{N996I/loxP\text{-}control}$) and corrected (LQT2-hiPSCs^{corr}) patient-specific hiPSCs (**B**) Bar graphs representing normalised field potential (FP) change induced by 30 µM E-3041 in mutated vs. corrected LQT2-hiPSCs. (**C**) FPD dose response relationship in presence of increasing amounts of E-4031 in wild-type and mutated hESCs (*NKX2.5*$^{eGFP/w}$ hESCs and LQT2-hESCs^{N996I}, respectively). (**D**). Bar graphs representing normalised field potential (FP) change induced by 30 µM E-3041 in mutated vs. wild-type hESCs. * indicates statistical significance (P<0.05; two-way rmANOVA).

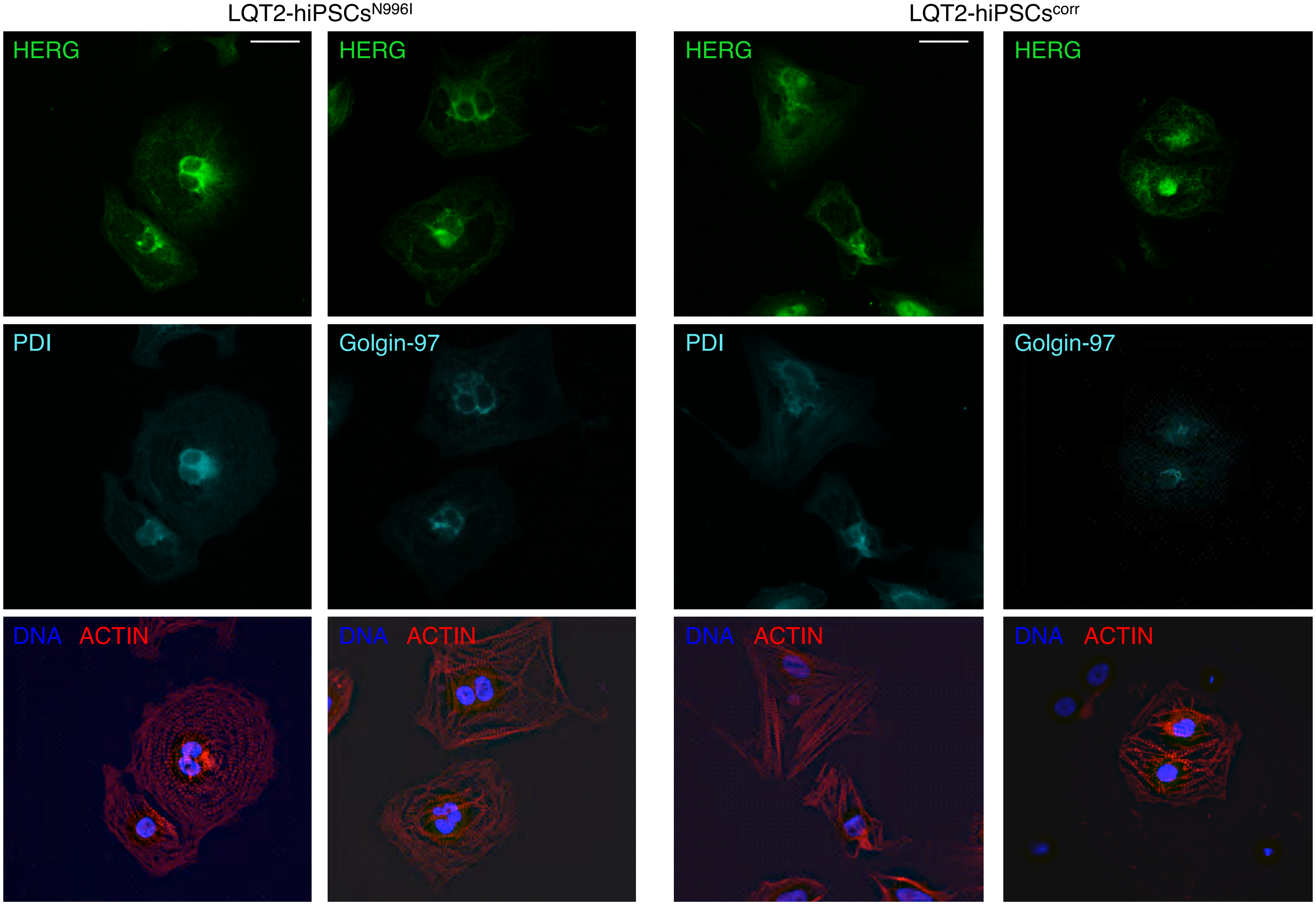

Supplementary Figure S8. HERG channel partially overlaps with ER and Golgi compartments in human CMs derived from mutated and corrected LQT2-hiPSCs. Immunofluorescence images of HERG channel (green) and of either the ER marker protein disulfide isomerase (PDI, cyan) or the Golgi compartment marker Golgin-97 (cyan) in representative human CMs derived from mutated and corrected LQT2-hiPSCs. Nuclei are stained in blue and ACTIN is shown in red by phalloidin stain. Scale bar: 25 μm.

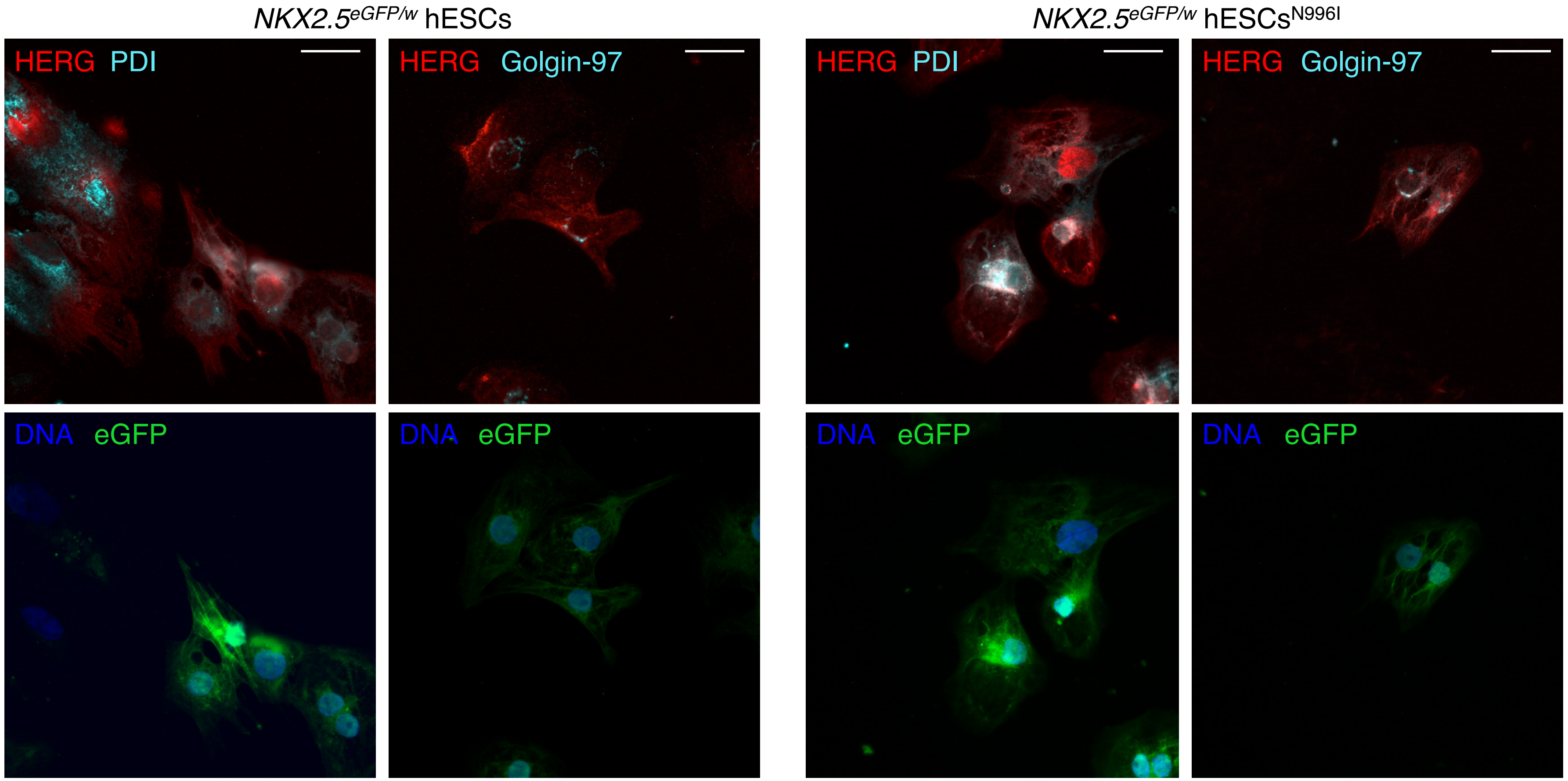

Supplementary Figure S9. HERG channel partially overlaps with ER and Golgi compartments in human CMs derived from wild-type and mutated hESCs. Immunofluorescence images of HERG channel (red) and of either the ER marker protein disulfide isomerase (PDI, cyan) or the Golgi compartment marker Golgin-97 (cyan) in representative human CMs derived from wild-type and mutated hESCs. Nuclei are stained in blue and eGFP is shown in green. Scale bar: 25 µm.

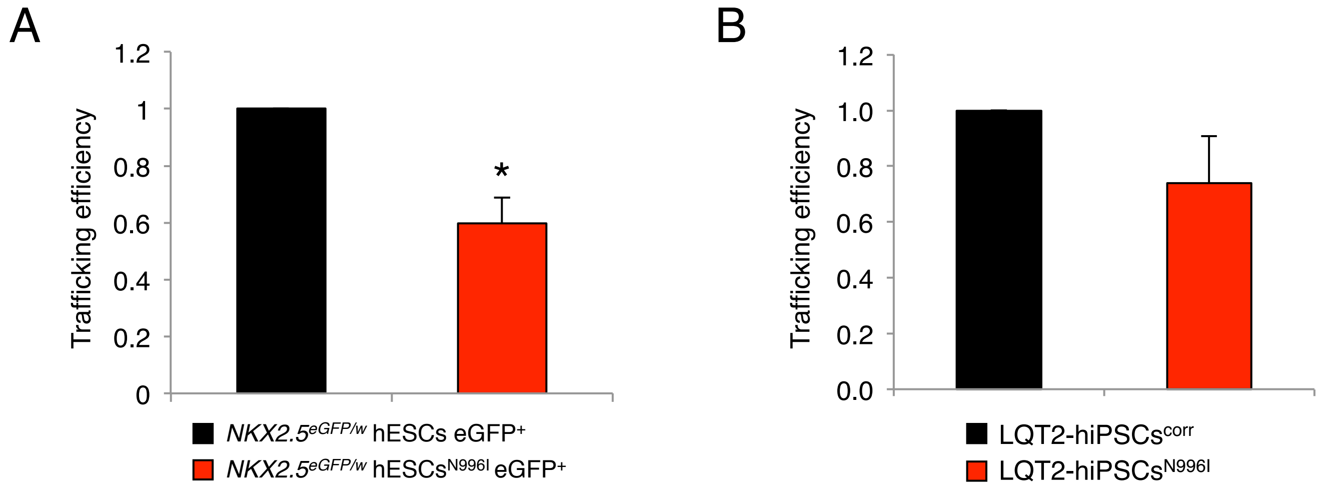

Supplementary Figure S10. Trafficking efficiency in hPSC-derived CMs. (**A**) Average trafficking efficiency in wild-type and mutated hESCs (*NKX2.5*$^{\mathrm{eGFP/w}}$ hESCs and *NKX2.5*$^{\mathrm{eGFP/w}}$ hESCs$^{\mathrm{N996I}}$, respectively) calculated from the densitometric analysis of Western blot experiments; values are presented as mean ±s.e.m., n=4. * indicates statistical significance (P=0.029, Mann-Whitney test) (**B**) Average trafficking efficiency in corrected and mutated hiPCs (LQT2-hiPCs^{corr} and LQT2-hiPSCs$^{\mathrm{N996I}}$, respectively) calculated from the densitometric analysis of Western blot experiments; values are presented as mean ±s.e.m., n=2. Trafficking efficiency=fg/(fg+cg), where fg=fully-glycosylated 155 kDa band and cg=core-glycosylated 135 kDa band.

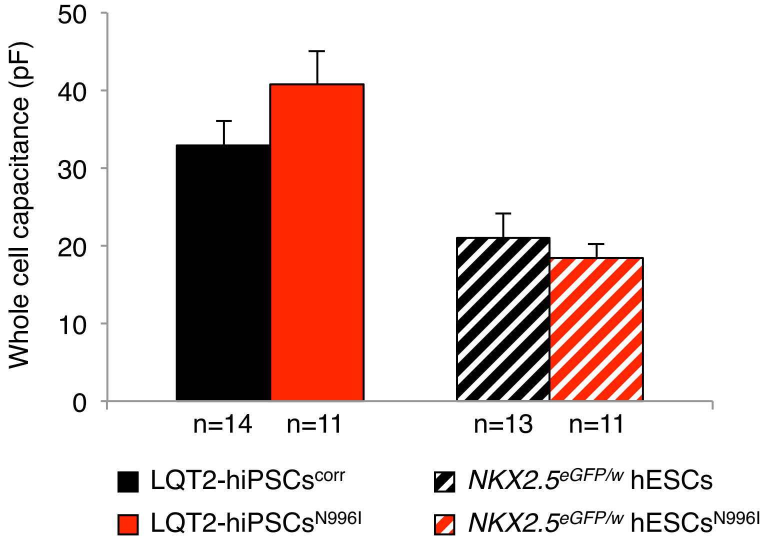

Supplementary Figure S11. Cell membrane capacitance in hPSC-derived CMs. The bar graph shows the average whole cell capacitance measured in corrected and mutated hiPSC-CMs (LQT2-hiPSC^{corr} and LQT2-hiPSC^{N996I}, respectively), and in wild-type and mutated hESCs (*NKX2.5*$^{eGFP/w}$ hESCs and *NKX2.5*$^{eGFP/w}$ hESCs^{N996I}, respectively). Values are presented as mean ±s.e.m. where the n. is indicated under each bar.
