# Supplementary figures and images for: Isogenic human pluripotent stem cell pairs reveal the role of a KCNH2 mutation in long-QT syndrome
Source: EMBO J. 2013 Nov 8;32(24):3161–75. doi: 10.1038/emboj.2013.240 (PMC3981141; doi:10.1038/emboj.2013.240)

Full unedited gel for Figure 2

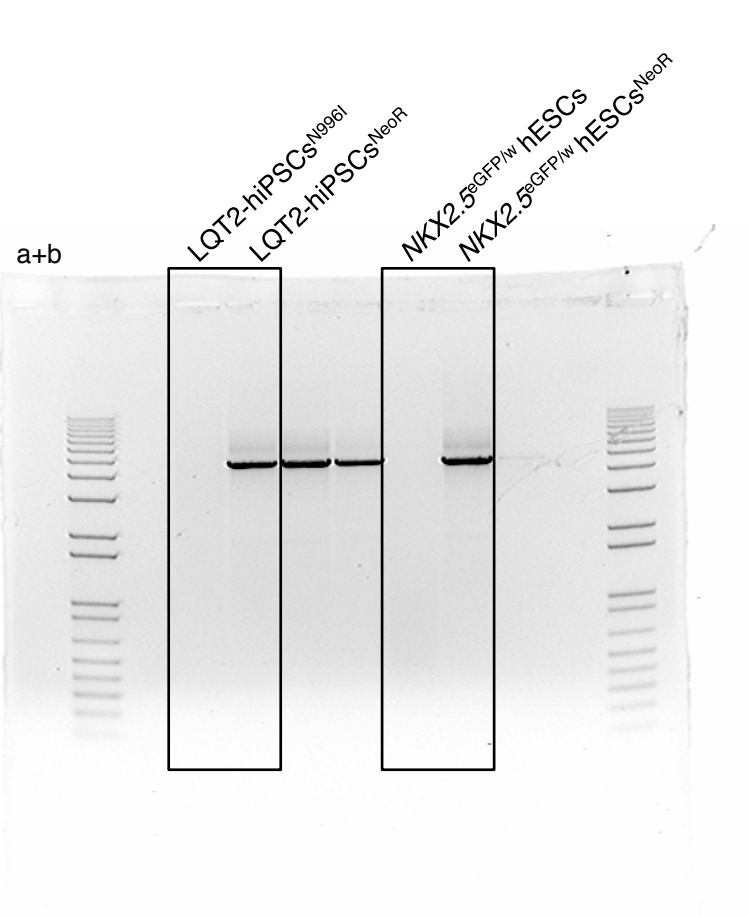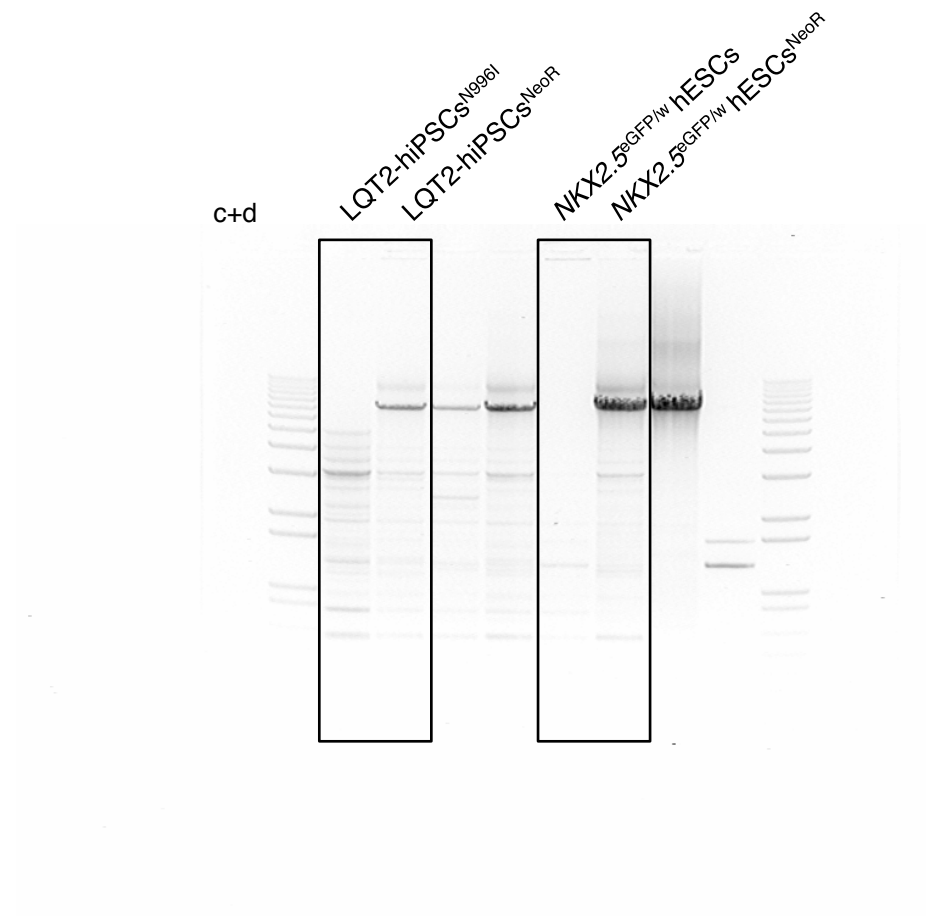

Supplement: Source Data for Figure 2 [file emboj2013240df2.pdf]
