# Supplementary material for: Isogenic human pluripotent stem cell pairs reveal the role of a KCNH2 mutation in long-QT syndrome
Source: EMBO J. 2013 Nov 8;32(24):3161–75. doi: 10.1038/emboj.2013.240 (PMC3981141; doi:10.1038/emboj.2013.240)

Full unedited Western Blots for Figure 8

<u>HERG in hESC-CMs #1 (for image and quantification in Figure 8)</u>

	NKX2.5$^{eGFP/w}$ hESCs		*NKX2.5$^{eGFP/w}$* hESCs^{N996I}	
HERG #1	eGFP^{+}	eGFP^{-}	eGFP^{+}	eGFP^{-}

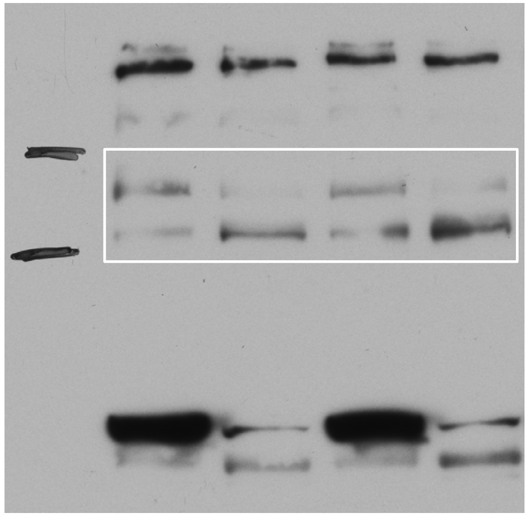

ACTIN #1

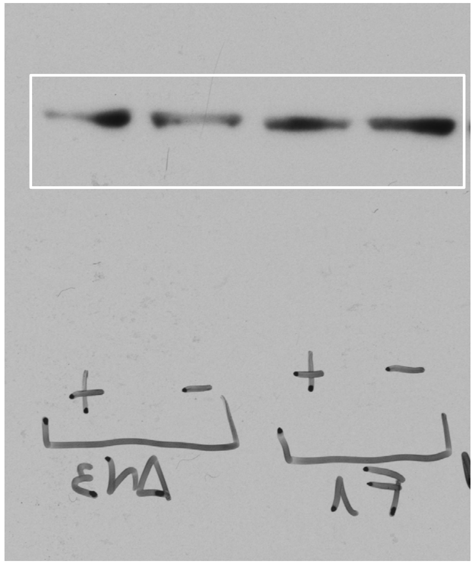

HERG in hESC-CMs #2 (for quantification in Figure 8)

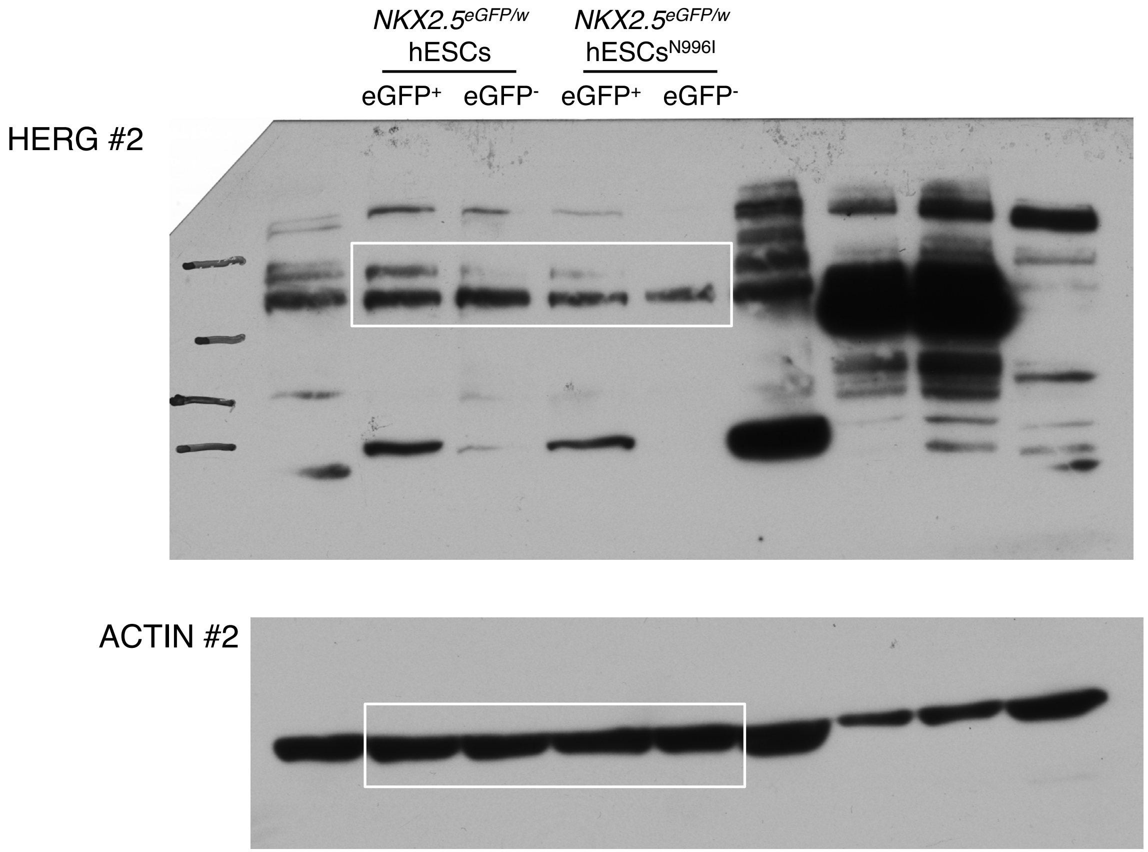

HERG in hESC-CMs #3 (for quantification in Figure 8)

NKX2.5$^{eGFP/w}$ hESCs		*NKX2.5*$^{eGFP/w}$ hESCs^{N996I}	
eGFP^{+}	eGFP^{-}	eGFP^{+}	eGFP^{-}

HERG #3

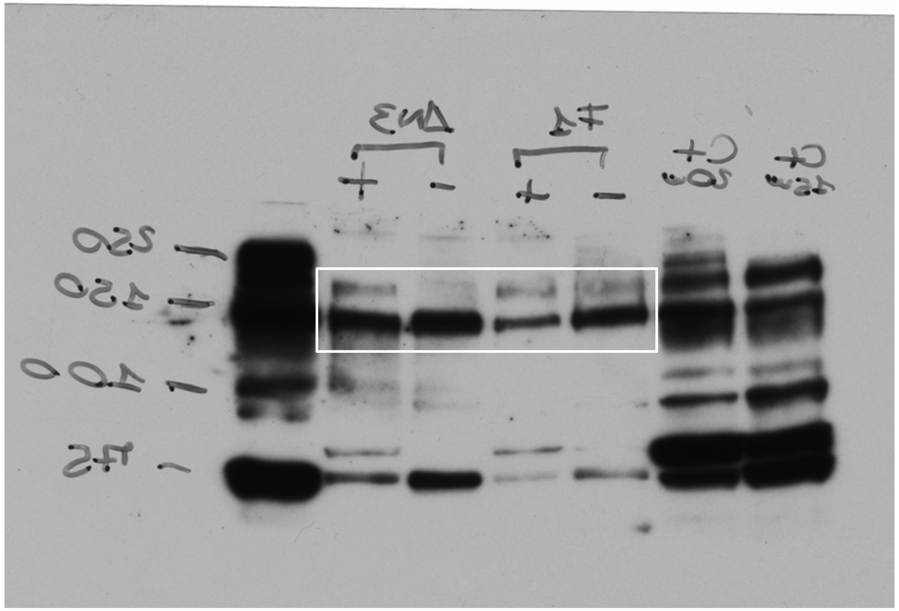

ACTIN#3

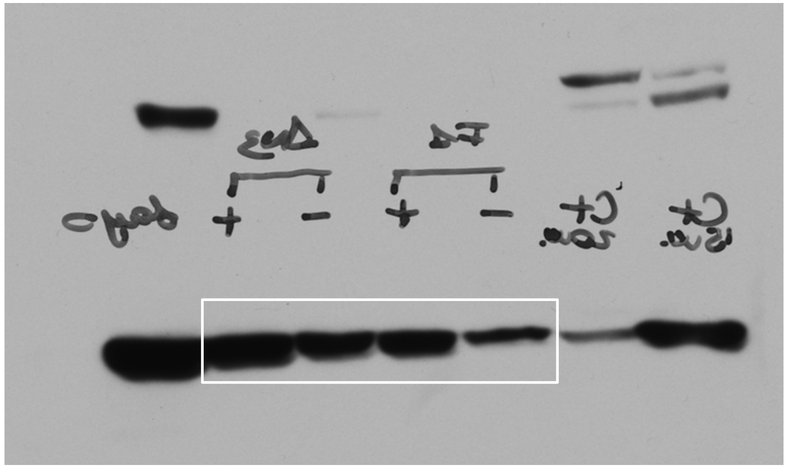

HERG in hESC-CMs #4 (for quantification in Figure 8)

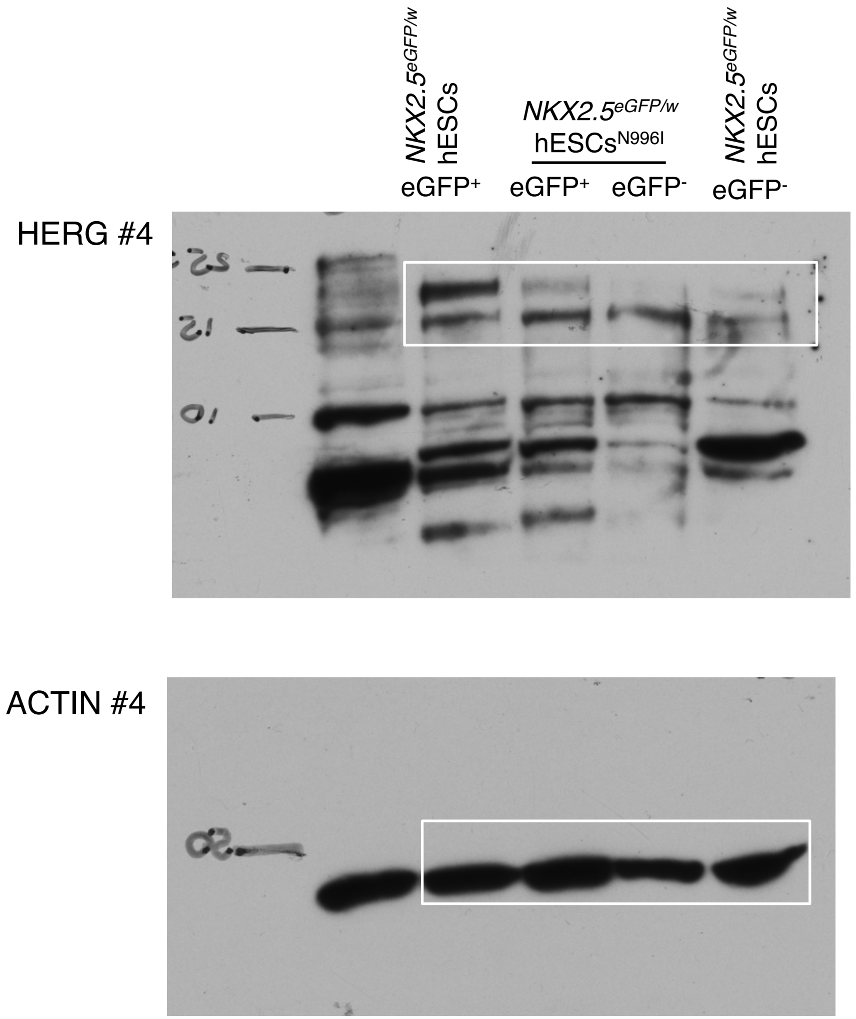

HERG in hiPSC-CMs #1 (for image and quantification in Figure 8)

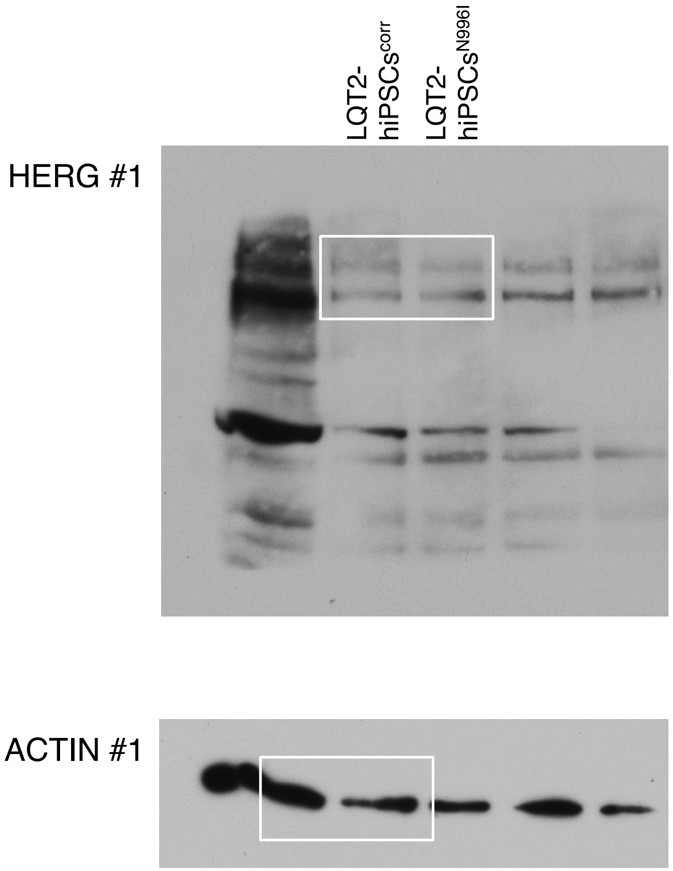

HERG in hiPSC-CMs #2 (for quantification in Figure 8)

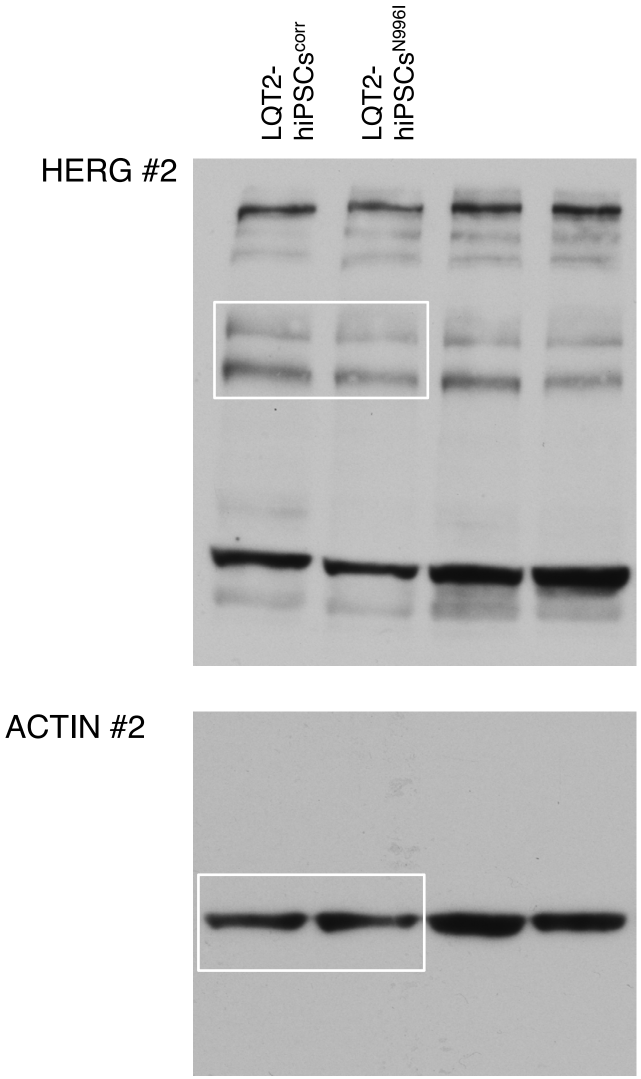

Supplement: Source Data for Figure 8 [file emboj2013240df8.pdf]
