# Supplementary material for: Isogenic human pluripotent stem cell pairs reveal the role of a KCNH2 mutation in long-QT syndrome
Source: EMBO J. 2013 Nov 8;32(24):3161–75. doi: 10.1038/emboj.2013.240 (PMC3981141; doi:10.1038/emboj.2013.240)

Full unedited Western Blots for Figure 9

<u>ATF6 in hESC-CMs (for Figure 9)</u>

ATF6

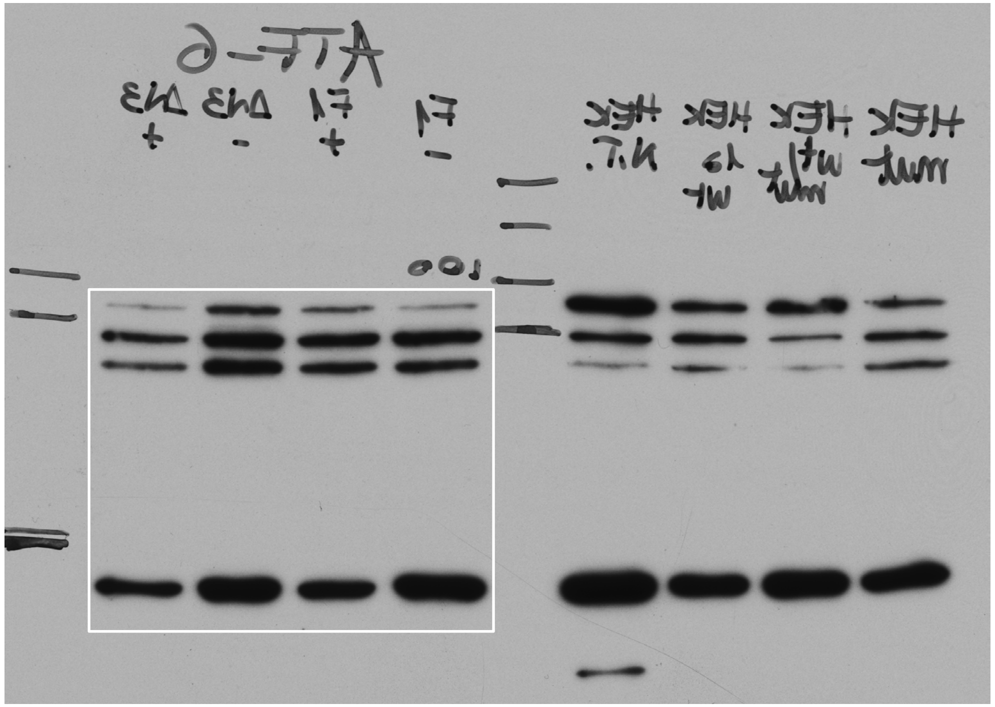

ACTIN

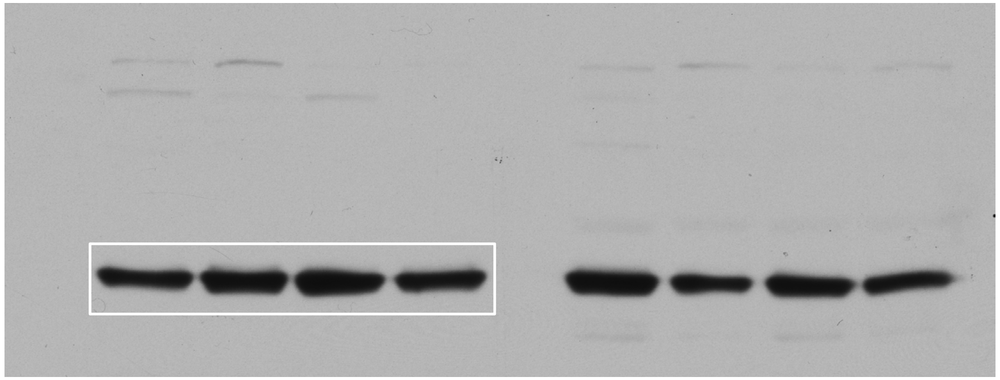

<u>CALNEXIN in hESC-CMs (for Figure 9)</u>

CALNEXIN

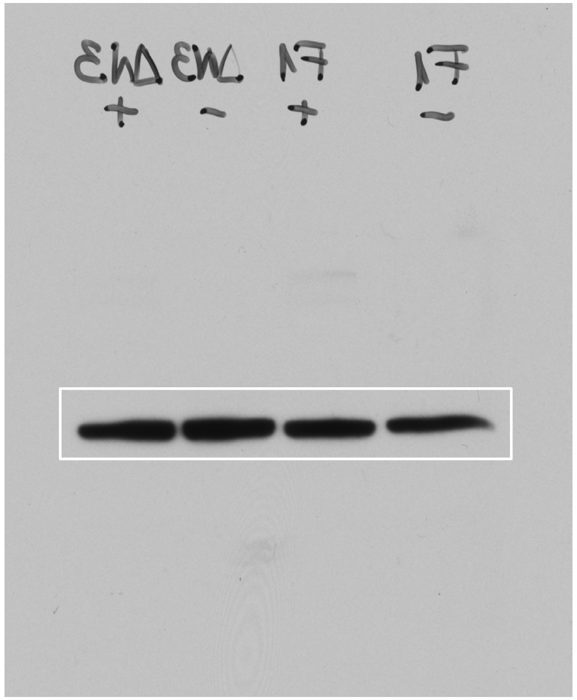

ACTIN

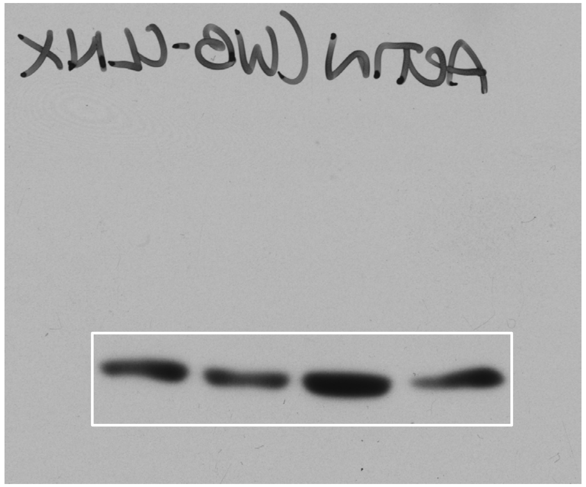

CALRETICULIN in hESC-CMs (for Figure 9)

CALRETICULIN

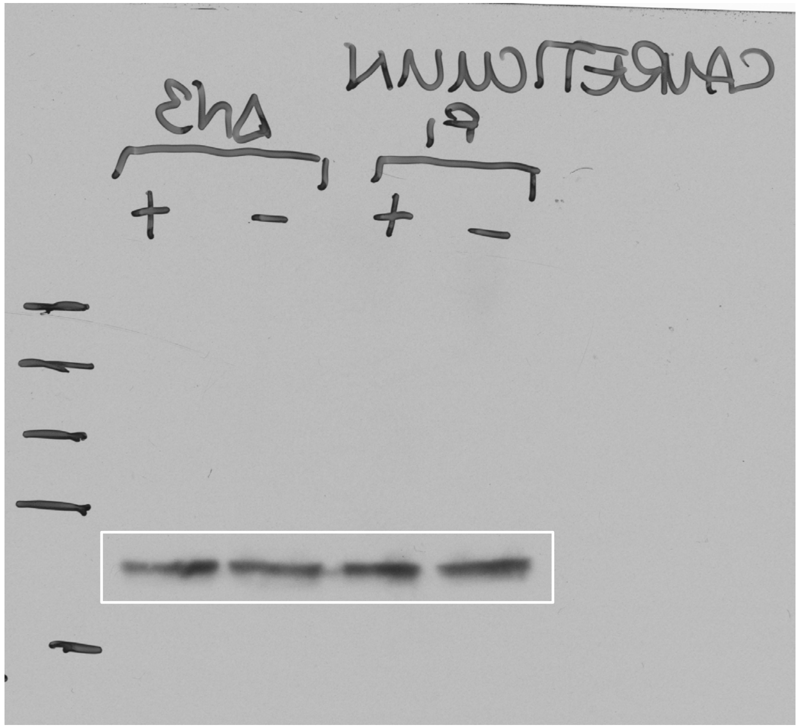

ACTIN

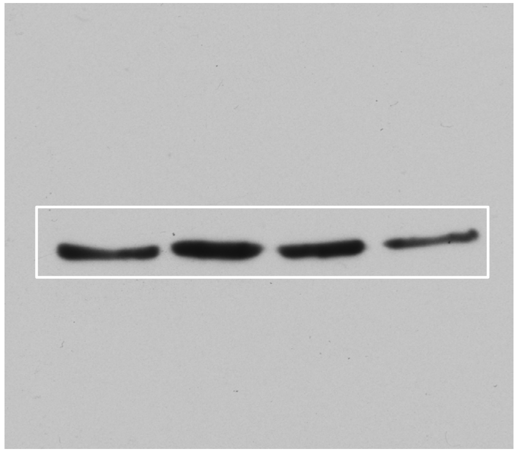

Inhibitors Treatment in hESC-CMs (for Figure 9)

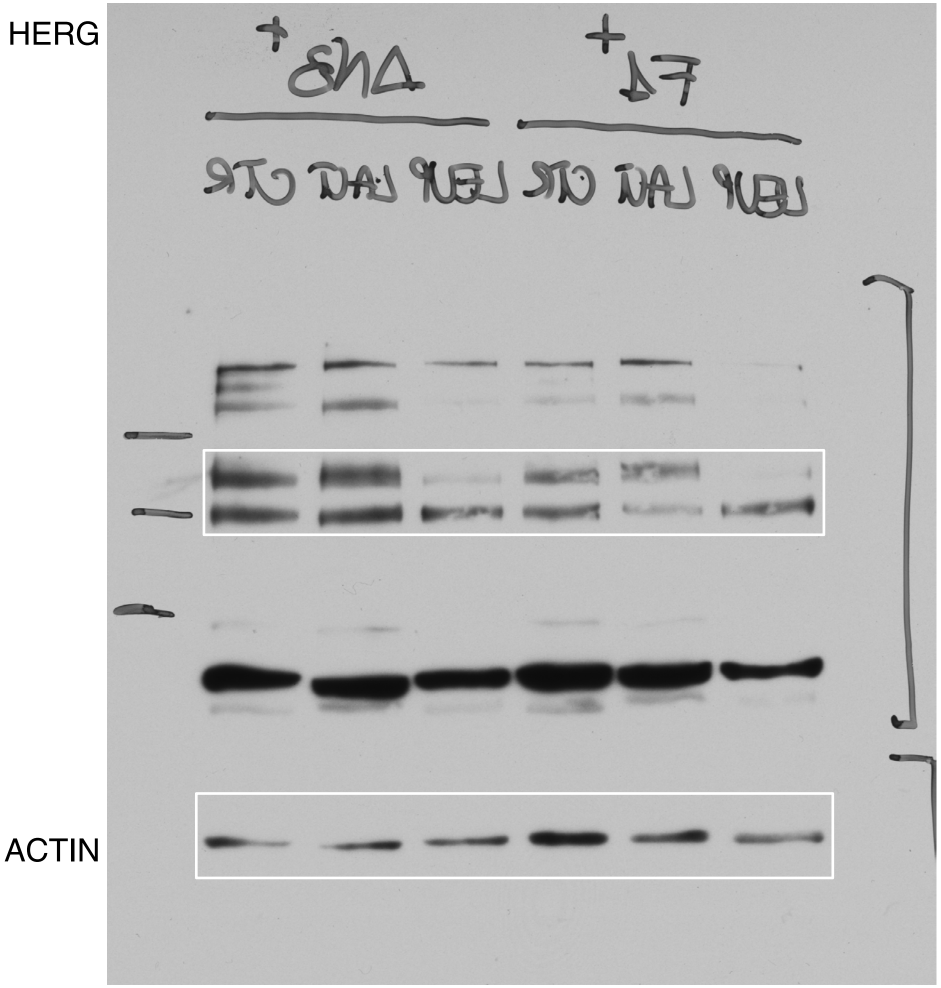

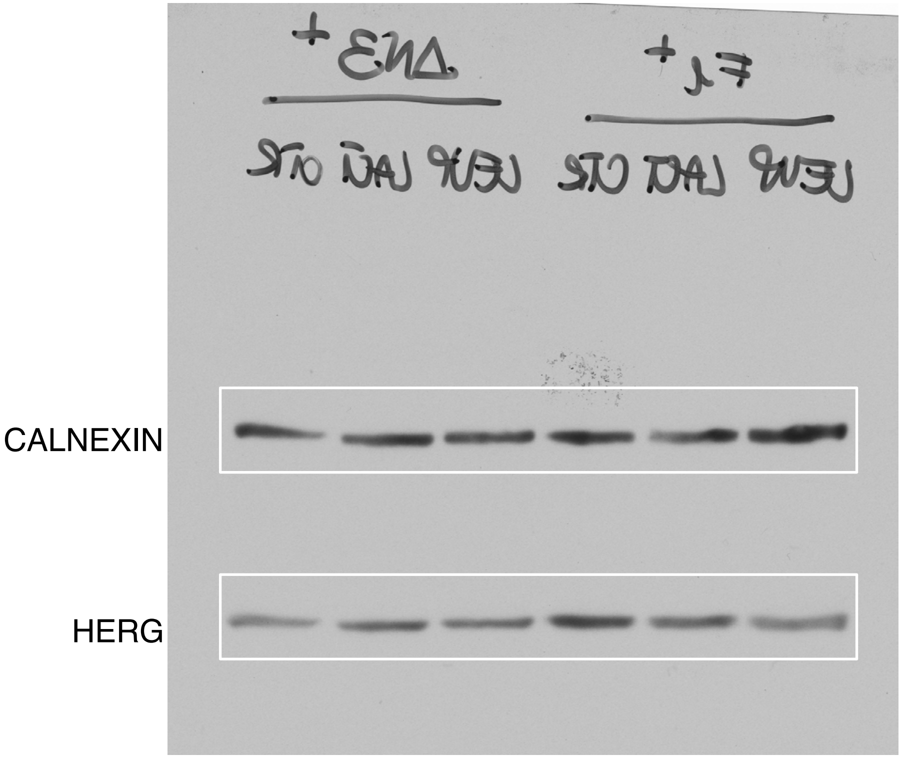

Supplement: Source Data for Figure 9 [file emboj2013240df9.pdf]
